# RNAi technology for management of banana bunchy top disease

**DOI:** 10.1002/fes3.247

**Published:** 2020-09-10

**Authors:** Temitope Jekayinoluwa, Leena Tripathi, Jaindra Nath Tripathi, Valentine Otang Ntui, George Obiero, Edward Muge, James Dale

**Affiliations:** ^1^ International Institute of Tropical Agriculture Nairobi Kenya; ^2^ Center for Biotechnology and Bioinformatics University of Nairobi Nairobi Kenya; ^3^ Department of Biochemistry University of Nairobi Nairobi Kenya; ^4^ Queensland University of Technology Brisbane Qld Australia

**Keywords:** banana, banana aphid, banana bunchy top disease, *banana bunchy top virus*, plantain, RNA interference

## Abstract

Banana bunchy top disease (BBTD) is one of the world's most destructive viral diseases of banana and plantain, causing up to 100% yield loss in severe cases. The disease is vectored by banana aphids (*Pentalonia nigronervosa*) and carried long distances through the movement of infected plant materials. The banana aphids harboring *banana bunchy top virus* (BBTV) present in banana producing regions are the sole vector and the most efficient method of transmitting the virus to the healthy plants. Controlling the spread of BBTD has been very challenging since no known banana germplasm is immune to BBTV. The disease can be managed with the use of virus‐free planting material and roguing. However, once BBTD is established in the field, it is very difficult to eradicate or manage it. Therefore, a more sustainable way of controlling the disease is developing host plant resistance against the virus and the vector. Biotechnological strategies via RNA interference (RNAi) could be used to target the banana aphid as well as BBTV to reduce virus‐associated yield losses of banana and plantain, which feed over 500 million people around the world. This review discusses the status of BBTD and perspectives on effective RNAi technologies for controlling BBTV and the vector, banana aphid, transmitting the virus as sustainable management of the disease.

## Graphical abstract

Banana bunchy top disease (BBTD) is one of the world's most destructive viral disease of banana and plantain causing up to 100% yield loss in severe cases. Biotechnological strategies via RNAi could be used to target banana aphid as well as__*banana bunchy top virus*__(BBTV) to reduce virus‐passociated yield losses of banana and plantain, which feed over 500 million people around the world. This review discusses the status of BBTD and perspectives on effective RNAi technologies for controlling BBTV and the vector, banana aphid, transmitting the virus as management of the disease.

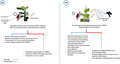

## 1 INTRODUCTION

Banana (*Musa* spp.), including plantain, is among important food security crops grown in over 136 subtropical and tropical countries, feeding over 500 million people (FAOSTAT, 2018). In terms of production, it is ranked the world's number one fruit crop (FAOSTAT, 2018). It serves as a major staple food in many parts of the world, especially in tropical countries. The worldwide production of bananas and plantains is over 155 million tons, with Africa accounting for over 44 million tons (FAOSTAT, 2018). India is the largest producer of banana generating over 30 million tons, while Central and West Africa is the most significant plantain growing region, accounting for about 19 million tons (FAOSTAT, 2018). This crop is mainly produced by smallholder farmers for consumption and as a major source of livelihood. Banana production generates an estimated income value of about USD 8 billion per year (FAO, 2019). A wide array of products (such as flour, juice, chips, wine) and parts of bananas and plantains (e.g., fruit, flower, pseudostem, fruit peel, corm, and leaf) are used as food, feed, and for medicinal purposes (Adeniji, Tenkouano, Ezurike, Ariyo, & Vroh‐Bi, 2010; Okareh, Adeolu, & Adepoju, 2015). Despite their market value and importance in ensuring global food security, insect pests such as banana weevil and nematodes, diseases like Fusarium wilt, black Sigatoka, banana Xanthomonas wilt, moko disease, banana bunchy top disease (BBTD), and banana streak disease, and other abiotic factors have been implicated in their declining yields (Chabi et al., 2018; Tripathi, Ntui, & Tripathi, 2019). All these aforementioned factors have remained a constant hindrance in achieving the full potential of bananas and plantains in supporting global food security.

**FIGURE 1:**
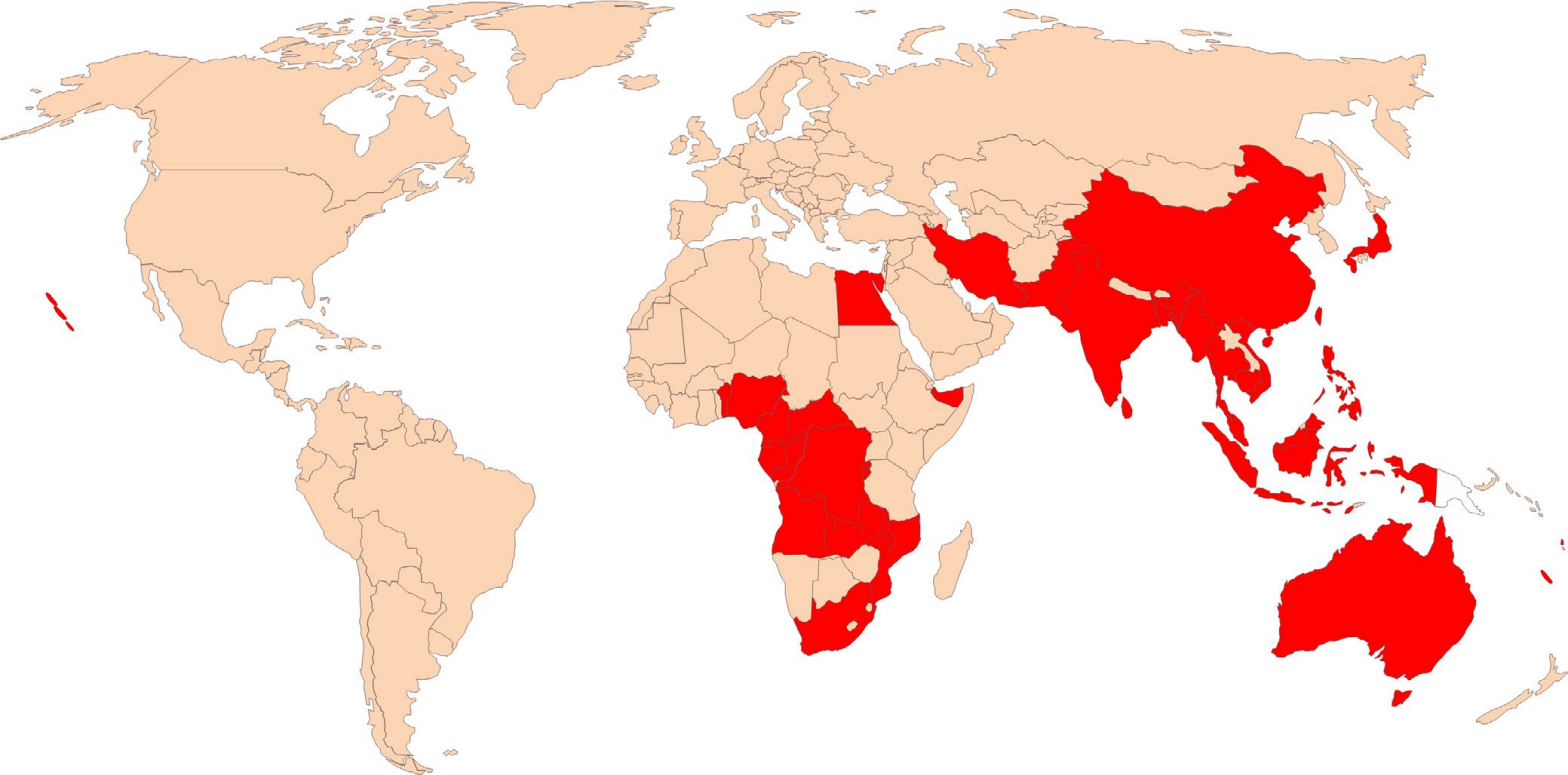
Global distribution of Banana Bunchy Top Disease (BBTD). Red color represents countries where BBTD has been reported

Banana bunchy top disease (BBTD) has been identified as one of the main biotic and economically important constraints to banana and plantain production worldwide (Dale, 1987). It is ranked as one of the world's 100 invasive alien species (Lowe, Browne, Boudjelas, & De Poorter, 2000), posing a significant negative economic influence on banana and plantain production. The disease is caused by banana *bunchy top virus* (BBTV, genus *Babuvirus*) and transmitted by banana aphids (*Pentalonia nigronervosa*) and the transboundary exchange of infected planting materials. BBTD was first reported in the 1880s in Fiji (Magee, 1927) and has since been spreading to other banana producing countries in the world including several African countries, Oceania, Asia, and South Pacific (Figure 1; Adegbola, Ayodeji, Awosusi, Atiri, & Kumar, 2013; Jooste, Wessels, & Van der Merwe, 2016; Kagy, Thomas, Sharman, & Mademba‐Sy, 2001; Kenyon, Brown, & Khonje, 1997; Khalid & Soomro, 1993; Kumar et al., 2011; Lokossou et al., 2012; Oben et al., 2009; Xie & Hu, 1995). In Africa, BBTD has spread to 17 different countries, and neighboring banana producing countries are at a high risk of being affected (Adegbola et al., 2013; Blomme et al., 2013; Jooste et al., 2016). In 2018, an incidence of BBTV was reported in Togo, although immediate technical action was taken to control its spread (IITA News, 2019). This is an indication of a continuous spread of the disease in banana producing regions causing decreased production of bananas and plantains; for example, 80% of the banana producing area was affected by BBTD in Malawi, and 88% reduction in banana cultivation from the 1970s to 2015 in India (Elayabalan, Subramaniam, & Selvarajan, 2015). Fruit production in infected plants reduces by 70%–100% within one season, and plantations are beyond recovery. The losses are not limited to a decrease in yields but also indirect production losses caused by the abandonment of susceptible but otherwise high yielding cultivars by farmers.

Plant resistance is the most appropriate method to control BBTD. There is no identified immunity to BBTV in the *Musa* germplasm. Biotechnological approaches could offer a pathway to improving and/or activating the defense mechanism of plants to diseases. The use of advanced biotechnological approaches such as host plant‐mediated RNA interference (RNAi) against BBTV and banana aphids has potential for the management of BBTD. The RNAi strategy has been demonstrated for the control of several viruses and insect vectors (Tables 1 and 2). This article presents an overview of recent progress and prospects in applying RNAi technology for control of BBTV and the vector, banana aphid, transmitting the virus as management of the disease.

**TABLE 1:** Application of RNAi for virus control in various crops

Virus	Family	Target viral gene	Crop	Phenotype	Reference
*Potato virus* Y	*Potyviridae*	*Protease*	*Nicotiana tabacum*	Immunity	Waterhouse, Graham, and Wang (1998)
*Bean golden mosaic virus*	*Potyviridae*	*AC1*	*Phaseolus vulgaris*	Resistance	Bonfim, Faria, Nogueira, Mendes, and Aragão (2007)
*Cucumber mosaic virus*	*Bromoviridae*	*RD1, RD6*	*Arabidopsis thaliana*	Immunity	Wang et al. (2010)
*Rice stripe Tenuivirus*	*Phenuiviridae*	*AGO 18*	*Oryza sativa*	Resistance	Wu et al. (2015)
*Banana Bunchy Top Virus*	*Nanoviridae*	*DNA‐R*,*BBTV viral genome*	*Musa* spp.	Resistance/tolerance	Elayabalan et al. (2013), Elayabalan et al. (2017), Krishna et al. (2013) and Shekhawat et al. (2012)
*Rice dwarf virus*	*Reoviridae*	*Pns12*	*Oryza sativa*	Resistance	Shimizu, Yoshii, Wei, Hirochika, and Omura (2009)
*Turnip yellow mosaic virus, Turnip mosaic virus*	*Tymoviridae, Potyviridae*	*amiR‐P69 159; amiR‐HC‐Pro 159*	*A. thaliana*	Resistance	Niu et al. (2006)
*Africa cassava mosaic virus*	*Germiniviridae*	*AC1*	*Manihot esculenta*	Resistance	Vanderschuren, Alder, Zhang, and Gruissem (2009)
*Cassava brown streak virus, Cassava brown streak Uganda virus*	*Potyviridae*	*Coat protein*	*M. esculenta*, *Nicotiana benthamiana*	Resistance	Patil et al. (2011) and Yadav et al. (2011)
*Alfalfa mosaic virus, Bean pod mottle virus, Soybean mosaic virus*	*Bromoviridae, Secoviridae, Potyviridae*	*Replicase genes*	*Glycine max*	Systemic resistance	Zhang et al. (2011)
*Cucumber mosaic virus*	*Bromoviridae*	*CMV‐O*	*Solanum tuberosum*	Resistance	Ntui et al. (2013)
*Sri Lanka Cassava mosaic virus*	*Geminiviridae*	*SLCMV*	*Manihot esculenta*	Resistance	Ntui et al. (2015)

**TABLE 2:** RNAi applications for aphid control

Target gene for RNAi	Insect vector	Plant virus transmitted by vector	Crop	Mode of action	Reference
*Laccase 1*	*Sitobion avenae*	*Barley yellow dwarf virus*	*Triticum avestivum*	Iron metabolism and immunity	Zhang, Fan, Francis, & Chen, 2018)
*Alkaline phosphatase*	*Diuraphis noxia*	*Barley yellow dwarf virus, barley yellow mosaic virus*	*T. avestivum*	Aphid‐plant interaction	Cooper et al. (2010) and Cooper et al. (2011)
*Structural sheath protein (shp)*	*Sitobion avenae*	*Barley yellow dwarf virus*	*Hordeum vulgare*	Aphid‐plant interaction and reproduction	Abdellatef et al. (2015)
*Mp10, Mp42*	*Myzus persicae*	*Potato virus* X (PVX), *Potato leafroll virus*	*Nicotiana benthamiana*, *Nicotiana tabacum*	Fecundity	Bos et al. (2010)
*C002*	*Acyrthosiphon pisum*	*Bean yellow mosaic virus*	*Vicia faba*	Fecundity/survival	Mutti et al. (2008)
*Acetylcholine sterase*	*Myzus persicae*	*Potato virus* X (PVX), *Potato leafroll virus*	*N. tabacum*	Resistance	Guo, Song, et al. (2014)
*KAT, Pepck, Gp*	*Toxoptera citricida*	*Citrus tristeza virus*	*Citrus sinensis*	Wing development	Shang et al. (2016)
*FAD7*	*Myzus persicae*	*Potato virus* X (PVX), *Potato leafroll virus*	*Lycopersicon esculentum*	Resistance	Li et al. (2018)
*C002, Rack1*	*Myzus persicae*	*Potato virus* X (PVX), *Potato leafroll virus*	*N. benthamiana*, *Arabidopsis thaliana*	Fecundity	Pitino et al. (2011)
*Chitin synthase 1 (CHS1)*	*Sitobion avenae*	*Barley yellow dwarf virus*	*T. avestivum*	Fecundity	Zhao et al. (2018)
*MpC002, MpPlntO2 and Rack 1*	*Myzus persicae*	*Potato virus* X (PVX), *Potato leafroll virus*	*A. thaliana*	Celluar process, aphid‐plant interaction	Coleman, Wouters, Mugford, and Hogenhout (2015)
*Ap‐crt and Ap‐cath‐L*	*Acyrthosiphon pisum*	*Bean yellow mosaic virus*	*Pisium sativum*	Developmental stage	Jaubert‐possamai et al. (2007)

Note

Common names of aphids and plants; *Sitobion avenae*: Grain aphid, *Diuraphis noxia*: Russian wheat aphid, *Myzus persicae*: Green peach aphid, *Acyrthosiphon pisum*: Pea aphid, *Toxoptera citricida*: Brown citrus aphid, *Triticum avestivum*: Wheat, *Hordeum vulgare*: Barley, *Vicia faba*: Fava bean, *Lycopersicon esculentum*: Tomato, *Pisium sativum*: Pea.

## 2 BANANA BUNCHY TOP VIRUS

**FIGURE 2:**
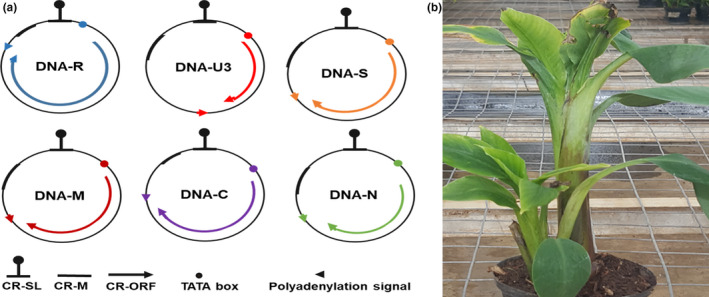
Genomic composition and effects of *Banana bunchy top virus* (BBTV) on the host plant. (a) Genomic organization of six ssDNA components of BBTV, (b) symptomatic banana plant showing stunted growth due to banana bunchy top disease

*Banana bunchy top virus* belongs to the genus *Babuvirus* and the family *Nanoviridae*. It is a complex circular single‐stranded DNA (ssDNA) virus that multiplies within the phloem tissue of a host plant (Mandal, 2010). The genome of BBTV is multipartite, comprising of six circular components (Figure 2) with an approximate size of 1.1 kb each (Burns, Harding, & Dale, 1995; Harding, Burns, & Dale, 1991; Harding, Burns, Hafner, Dietzgen, & Dale, 1993) and are known to promote the pathogenicity of the virus. They were initially named as DNA 1–6 but recently named as DNA‐R, DNA‐U3, DNA‐S, DNA‐M, DNA‐C, and DNA‐N, respectively (Abdel‐Salam, Dahot, & Sadik, 2012; Harding et al., 1991; Wu, You, & Soong, 1994). The six components are encapsidated within separate virions, each about 18–20 nm in diameter (Harding et al., 1993). All the six integral components have a common genome organization comprising of a major common region (CR‐M), stem‐loop common region (CR‐SL), potential TATA box 3’ of the stem‐loop, at least one open reading frame (ORF) for a major gene in the virion sense, and polyadenylation signals associated with each gene (Figure 2; Burns et al., 1995). The major component of BBTV DNA‐R encodes two open reading frames and other components encode one protein each (Beetham, Hafner, Harding, & Dale, 1997; Burns et al., 1995). Five of the six components have a large open reading frame in the virion sense and a stem‐loop structure in the noncoding intergenic region (Burns et al., 1995). The stem‐loop initiates replication of viral protein.

DNA‐R encodes a replication initiation protein (Rep) responsible for initiating viral DNA replication, DNA‐S encodes the coat protein (CP), DNA‐C encodes the cell‐cycle link protein (Clink), DNA‐M encodes the movement protein (MP), and DNA‐N encodes the nuclear shuttle protein (NSP), while the function of DNA‐U3 is unknown (Abdel‐Salam et al., 2012; Burns et al., 1995; Wanitchakorn, Hafner, Harding, & Dale, 2000; Wanitchakorn, Harding, & Dale, 1997). BBTV DNA‐R, DNA‐U3, DNA‐S, DNA‐M, DNA‐C, and DNA‐N (DNA‐1–DNA‐6) have been consistently associated with BBTV isolates globally, suggesting they are integral components of the BBTV genome.

*Banana bunchy top virus* has been distributed widely with confirmed infection in Africa, Asia, Australia, and South Pacific islands but significantly not in the Americas (Figure 1). There are two broad groups of BBTV isolates identified based on nucleotide sequence differences between their genome components and geographical delineation (Karan, Harding, & Dale, 1994; Kumar et al., 2011). The “South Pacific” group comprises isolates from Australia, Bangladesh, India, Myanmar, Pakistan, Sri Lanka, Fiji, Western Samoa, Tonga, Hawaii, and Africa, while the “Asian” group comprises isolates from China, Indonesia, Japan, Philippines, Taiwan, Thailand, and Vietnam (Karan et al., 1994; Kumar et al., 2011). Both groups differ from each other, with an average of 9.6% (DNA‐R), 11.86% (DNA‐S), and 14.5% (DNA‐N) over the entire nucleotide sequence. However, the difference in the major common region (CR‐M) between the two groups is about 32% (DNA‐R), 38.6% (DNA‐S), and 27% (DNA‐N; Karan et al., 1994).

## 3 TRANSMISSION AND SPREAD OF BBTV

*Banana bunchy top virus* is transmitted by the banana aphid in a circular, nonpropagative, and persistent manner (Di Mattia et al., 2020; Watanabe, Borthakur, & Bressan, 2016). This implies that the virus does not replicate within the vector and can persist in the vector for its whole lifespan. The aphid acquires the virus after at least four hours of feeding on an infected plant and retains BBTV throughout its adult life (15–20 days) but does not transmit to its progeny (Nelson, 2004). The virus transmission efficiency for individual aphids is about 46%–67%, and the nymphs acquire the virus more efficiently compared with adult aphids (Magee, 1927). The spread of disease by aphids is only short distance, within the 20 m of the nearest source of infection (Allen, 1978).

The winged aphids that often develop after 7–10 generations of wingless individuals are most likely responsible for the rapid spread of the virus (Nelson, 2004; Young & Wright, 2005). These winged aphids can transmit the virus to a healthy banana plant by feeding on it for as long as 15 min to about 2 hr (Dale, 1987; Hu, Wang, Sether, Xie, & Leonhardt, 1996). Both wingless and winged aphids are able to transmit viruses. There are two major variants of *Pentalonia* species (*P. nigronervosa* and *Pentalonia caladii*) found on various host plants such as ginger, tomatoes, taro, *Xanthosoma* spp., cardamom, *Heliconia* spp., calla, and costus (Duay, Miller, Wall, Pike, & Foottit, 2014; Pinili, Nagashima, Dizon, & Natsuaki, 2013; Watanabe, Greenwell, & Bressan, 2013). However, the preferred hosts for *P. nigronervosa* (banana aphid) are the *Musa* species and *Ensete* (Robson, Wright, & Almeida, 2007).

Aphids ingest the BBTV with the sap of the infected plants, which are internalized and cross their gut cells, and then translocated to the hemocoel (Watanabe & Bressan, 2013). Upon feeding on the infected plant, BBTV translocates through the aphid vector rapidly. It is internalized into the anterior midgut where it accumulates and is retained at concentrations higher than the hemolymph or the salivary glands (Watanabe & Bressan, 2013). The ingested virus passes through the gut epithelium, hemolymph, and salivary gland, thus establishing a specific virus–vector interaction (Brault, Uzest, Monsion, Jacquot, & Blanc, 2010). Once the virus is internalized within the salivary glands, the virions can be discharged to the plant tissues along with the saliva during the feeding process.

As BBTV is a multipartite virus, the infected plant should have a mixed virus population with six types of viral particles containing a distinct genome segment. At least one functional particle of each kind of viral particle must be transmitted for the successful passage of the integral genome to a new host plant (Iranzo & Manrubia, 2012). It is also possible that all the genome segments of the virus do not exist together in individual plant cells suggesting that the infection proceeds within the host plant through functional complementation of the distinct genes across distinct cells (Di Mattia et al., 2020). It was previously evident where only the coat protein was monitored in studies tracking the BBTV within its aphid vector (Watanabe et al., 2016; Watanabe & Bressan, 2013). It is unknown whether nanoviruses invade individual vector cells with a small or large number of virus particles, allowing the distinct genome segments to travel altogether or separately from gut to salivary glands (Di Mattia et al., 2020). It was recently demonstrated that the success of nanovirus–vector interaction depends on a nonstructural helper component that is shown to be mandatory for viral accumulation within the gut cells (Di Mattia et al., 2020). The viral protein nuclear shuttle protein (NSP) encoded by DNA‐N has been identified as a helper factor essential for aphid viral transmission of nanoviruses (Gaafar & Ziebell, 2019; Grigoras et al., 2018). Di Mattia et al. (2020) demonstrated the colocalization of both the NSP and CP with the viral genome segments. This suggests that NSP–virus particle complexes are the viral form that cycles within the aphid body. An exclusion of DNA‐N from the eight components of a nanovirus, *Faba bean necrotic stunt virus* (FBNSV), genome prevented transmission of the virus by an aphid (Gaafar & Ziebell, 2019). Furthermore, altering the nuclear shuttle protein of FBNSV, by including nonfunctional proteins to the carboxy‐terminus led to an infectious but nontransmissible virus (Grigoras et al., 2018). This helper factor makes it possible for the virus to bind receptors within the aphid's stylet to facilitate transmission (Brault et al., 2010; Gaafar & Ziebell, 2019). The stylets of aphids have a unique ability to bind, retain, and release the virus into plant phloem during feeding (Deshoux et al., 2020).

Upon infection, the virus replicates rapidly within the phloem, and the plant develops disease symptoms. The morphology of the banana plant is distorted, leading to symptoms such as curling and shriveling of leaves, stunted growth, and in severe cases may lead to plant death (Figure 2; Elayabalan et al., 2015). The transmission efficiency of BBTV via banana aphid depends mostly on the viruliferous potential of the aphids (Hafner, Harding, & Dale, 1995), temperature (Anhalt & Almeida, 2008; Barton & Ives, 2014), and vector life stage and plant access period (Wu & Su, 1990).

Besides transmitting the virus, the banana aphids acquire sugars while feeding on the nutrients from phloem cells of the banana plant and excrete honeydew, which promotes the growth of sooty mold fungi and serve as ants feed. This could hinder photosynthetic activities of the plant and could as well lead to wilting and stunting, especially for younger plants (Figure 2). Infected planting material such as suckers for vegetative propagation can be an alternate source of spreading the disease, especially to long distances when germplasms are exchanged across borders or regions without adequate diagnostics and/or quarantine measures (Dale, 1987; Kumar et al., 2011). Since banana aphids are present in all banana producing countries, the propagation of infected planting materials provides an avenue for banana aphid vector to transmit the virus.

## 4 CONVENTIONAL METHODS OF CONTROLLING BBTD AND ASSOCIATED CHALLENGES

Several strategies have been employed in managing the spread of BBTD; however, these methods have limitations. Rouging is a convenient and common means of eradicating the infected plant (van den Bosch, Jeger, & Gilligan, 2007; Sisterson & Stenger, 2013); however, if it is not done correctly, the aphids could migrate from the diseased plant and infect surrounding healthy plants. Chemical control strategy through the use of organophosphate insecticides such as diazinon, imidacloprid, and paraffinic oil has been employed to control the spread of banana aphids (Robson et al., 2007). However, the applied chemicals may not reach aphids in the inner sections of the plant, such as the inner part of the cigar leaf and within leaf sheaths of the pseudostem. Chemicals may also have adverse effects on the applicator, besides killing other off‐target species. Mortality of the aphids depends not only on the treatment concentration but also on the age of the leaf as imidacloprid is not potent on young leaves (Robson et al., 2007) besides not being cost‐effective. Biopriming, an alternative treatment to pesticides, uses beneficial microorganisms such as rhizobacteria and/or endophytic bacteria. Although, there was a reported reduction in BBTV incidence under field conditions, it could not provide absolute resistance to BBTD (Kavino et al., 2007). Consequently, host plant resistance is always considered as the potentially viable option for control of the viral disease.

Conventional breeding has long been a useful tool for developing disease‐resistant material. The success of traditional breeding depends mainly on the availability of disease‐resistant cultivar(s), which may be used to create improved banana/plantain varieties. However, there is no known germplasm with immunity or high levels of resistance to BBTD. Although there has been variation in susceptibility among some cultivars in the banana germplasm (Ngatat et al., 2017), it remains challenging to identify a suitable source of resistance in germplasm that could be incorporated into a conventional breeding program.

Plant tissue culture techniques have significantly contributed to generating clean banana planting materials (Ramos & Zamora, 1990; Wang, Panis, Engelmann, Lambardi, & Valkonen, 2009) but does not guarantee absolute elimination of BBTD as clean plants can be reinfected if planted in an infected field with viruliferous aphids. Colonies of aphids can be found at the base of the pseudostem at soil level and several centimeters below the soil surface (Thomas, 2008), it is not unlikely that clean tissue culture banana/plantain planted close to an infected plant may be vulnerable to infection especially if a viruliferous aphid feeds on it.

## 5 ADVANCES IN BIOTECHNOLOGICAL APPROACHES FOR CONTROLLING THE SPREAD OF BBTV

Effective methods of controlling the spread of BBTD could be achieved by eradicating/ destroying the vector, *P. nigronervosa*, and preventing the vector from transmitting the viruses, protecting the crop, or using virus‐resistant varieties. Since BBTD‐resistant varieties are yet to be found in the banana germplasm, alternative means of protecting the crop by minimizing or eradicating the spread of BBTD needs to be explored.

There is a critical need to develop banana and plantain varieties with durable resistance to BBTV. Genetic modification is an attractive strategy to achieve this, and the technology to modify bananas is now advanced. Already there have been a number of successful field trials of disease resistance banana, particularly for bacterial wilt resistance (Tripathi et al., 2019). Also, the development of virus and vector resistant crops using RNAi is quite advanced (Tables 1 and 2).

## 6 RNAi‐BASED APPROACHES FOR VIRUS CONTROL

RNA interference targeting the viral genes is one of the potential immune response employed by plants to silence the expression of viruses upon attack. This mechanism, alongside other associated processes, has been studied and applied in developing disease‐resistant crops. RNAi acts by suppressing transcriptional process (TGS—transcriptional gene silencing) or activating degradation of sequence‐specific RNA (PTGS—post‐transcriptional gene silencing; Agrawal et al., 2004). The presence of a double‐stranded RNA (dsRNA) is a potent trigger and end product of RNAi, which keeps the cycle of silencing active in biological systems. When dsRNA is induced, an enzyme known as the dicer recognizes and cleaves the dsRNA into smaller RNA fragments of about ≈21–25 nucleotides known as the small interfering RNAs (siRNA; Agrawal et al., 2004). The populations of siRNA fragments are incorporated into a nuclease containing complex referred to as the RNA‐induced silencing complex (RISC) where it is bound to the Argonaute proteins, the siRNA unwinds, and one strand is discharged to reinitiate the process while the other binds with the homologous target mRNA and degrades or silence its expression (Waterhouse & Helliwell, 2003). The stoichiometry of the siRNA accumulation and the homologous mRNA specific‐sequence influences the efficacy of the RNAi mechanism. Another factor influencing the effectiveness of RNAi is the presence of suppressors, encoded as proteins in plants and viruses. They either prevent the production or accumulation of siRNA and downstream degradation of viral RNA or sequester siRNA, preventing them from being transported to RISC (Tenllado & Diaz‐Ruiz, 2001).

Almost all plant viruses encode viral suppressors of RNA silencing (VSRs), inhibiting the key steps of RNAi system to neutralize the RNAi‐based antiviral defense of the host plant. Several proteins such as V2 protein of the *Tomato yellow leaf curl virus* (TYLC) and *Tomato yellow leaf curl China virus* (TYLCCNV), AC2/AL2 protein of *Begomoviruses*, C2/L2 protein of *Curtoviruses*, betasatellites (βC1) of TYLCCNV, cysteine‐rich protein (CRP) and triple gene block protein 1 (TGBp1) of *Potato virus* *M* (PVM), helper component proteinase (HC‐Pro) of potyviruses such as *Turnip mosaic virus* (TuMV), *Tobacco etch virus* (TEV), 2b of *Cucumber mosaic virus* (CMV), P38 of *Turnip crinkle virus* (TCV), P19 of *Cymbidium ring spot virus* (CRSV), viral genome‐linked protein (VPg) of potyviruses, P1 helper component proteinase (P1/HC‐Pro) of potyviruses, P6 of *Cauliflower mosaic virus* (CaMV), CP and P6 of *Olive mild moaic virus* (OMMV), and MP of babuviruses, dianthoviruses and citriviruses are some of the examples of VSRs that have been identified to inhibit RNA silencing (Anandalakshmi et al., 1998; Buchmann, Asad, Wolf, Mohannath, & Bisaro, 2009; Cheng & Wang, 2017; Csorba, Kontra, & Burgyan, 2015; Li, Huang, Li, & Zhou, 2014; Senshu et al., 2011; Varanda et al., 2018; Zrachya et al., 2007). One of the approaches for controlling viral diseases is to develop RNAi‐based viral resistance in the transgenic host plants by inactivating these VSRs.

The CP and MP of BBTV encoded by DNA‐S and DNA‐M have been identified as suppressors of RNAi (Niu et al., 2009). It was demonstrated that CP and MP act as suppressors at different steps in the RNAi silencing pathways. MP was found to be a more robust suppressor of RNAi compared with CP. Further, Amin et al. (2010) detected MP and the cell‐cycle link protein (Clink) encoded by DNA‐C as suppressors of RNA silencing. MP could be considered a potential candidate for developing resistance against BBTV using the RNAi approach. Besides acting as a suppressor of RNA‐mediated gene silencing, MP allows an efficient cell to cell propagation, by dodging the host cell wall barrier. BBTV is shuttled out of the nucleus by nuclear shuttle protein (NSP), and MP transports the DNA‐NSP complex to cell plasmodesmata and facilitates further movement across the cell wall. Thus, inhibition of MP can provide strong resistance to BBTV. Therefore, identifying other RNA‐silencing suppressors of BBTV, understanding the cross‐talk between the host–pathogen interaction, biochemical components, and subsequently interfering with the suppressors can improve the efficiency of RNAi for virus control in host plants.

Nevertheless, RNAi has proven to be a useful approach in preventing the expression of viruses in plants (Table 1). It has been successfully used in conferring substantial varying level of resistance to plant viruses such as *Mungbean yellow mosaic India virus*, *Papaya ringspot virus*, *Soybean mosaic virus*, *Cucumber mosaic virus*, and *Cassava mosaic virus* (Jia et al., 2017; Kumar, Tanti, Patil, Mukherjee, & Sahoo, 2017; Ntui et al., 2014, 2015; Thu et al., 2016). Similar promising results have been obtained in suppressing the expression of BBTV. Krishna et al. (2013) targeted four viral BBTV components (DNA‐R, DNA‐S, DNA‐M, and DNA‐C) through RNAi and achieved partial resistance to BBTV in transgenic Grand Nain banana under controlled conditions. Likewise, targeting DNA‐R in hill banana resulted in symptomless plants with suppressed symptoms for BBTD (Elayabalan et al., 2013; Elayabalan, Subramaniam, & Selvarajan, 2017). The generation of BBTV‐resistant plants using RNAi and intron‐hairpin‐RNA (ihpRNA) transcripts corresponding to the DNA‐R, established the efficacy of RNAi mechanism in developing BBTV‐resistant lines (Shekhawat, Ganapathi, & Hadapad, 2012). In that study, RNAi was applied for developing resistance against BBTV targeting the full coding sequence of the replication protein gene (*Rep*) or partial coding sequence of *Rep* gene together with its 5′ partial upstream regulatory region of the BBTV. The transgenic plants showed resistance against BBTV up to 6 months postinoculation with viruliferous aphids. The prospect of RNAi relies on a comprehensive understanding of the multipartitism of BBTV and its synergistic interaction with its host and downstream biochemical machinery, which would elucidate how to improve the specificity of RNAi for virus transmission control.

## 7 RNAi‐BASED APPROACH FOR INSECT VECTOR CONTROL

*Banana bunchy top virus* is very difficult to manage, and not much success has been achieved toward developing host plant resistance through breeding or transgenic approach. Therefore, controlling virus transmission by aphids represents a new alternative for the management of aphid‐borne BBTD. One option could be to block the aphid‐mediated transmission of the virus by interfering with aphid–virus interactions to inhibit virus acquisition by aphids. The efficiency of virus acquisition is one critical parameter in determining aphid transmission efficiency. The aphid transmission can be blocked by impairing the virus acquisition at the gut epithelial cell barrier via direct interference with aphid–virus protein interactions. The receptors and/or virus‐binding protein(s) present on the gut and accessory salivary gland promote the transmission specificity (Brault et al., 2010). Therefore, the vector–virus protein interaction could be a potential target for RNAi by blocking the vector's ability to transmit the virus (Heck & Brault, 2018). The cuticular proteins play a crucial role in plant virus transmission by influencing the structure of cuticles, controlling virus–vector interaction, virus entry into the gut, and preventing virus degradation in the insect hemolymph (Deshoux, Monsion, & Uzest, 2018). A cuticular protein, receptor RR‐1, at the surface of the acrostylet in the pea aphid was identified and found to be associated with circulative virus transmission (Deshoux et al., 2018). The interaction between the nuclear shuttle protein encoded by DNA‐N and cuticular proteins in aphid's stylet may facilitate virus transmission. However, deactivating the helper factor and/or receptors by targeting *RR‐1* genes in banana aphid and DNA‐N using RNAi could be a potential way to abolish the transmission of the virus by the vector.

Another approach to control the apid‐transmitted virus is to reduce the vector population. Achieving this could require interfering with the vector's feeding machinery as most are phloem feeders and infect the host plant during feeding. RNAi is a feasible way of inducing a specific insecticidal effect on an insect vector without affecting off‐target organisms. RNAi has been successfully applied in insect orders like the coleopteran, lepidopteran, hemipteran insect pests (Baum et al., 2007; Knorr et al., 2018; Laudani et al., 2017; Poreddy, Li, & Baldwin, 2017; Yoon et al., 2018; Zha et al., 2011). Hemipteran insects such as whiteflies, and aphids are phloem feeders or sap‐sucking insects, damage the plant during feeding and act as vectors for transmitting the virus to the host plant (Chougule & Bonning, 2012). Managing the effect of these insect pests on plants via RNAi would call for identifying the critical gene(s) essential for the insect survival and/or adaptation on host plants. Identification of crucial genes for effective RNAi‐based control of aphids would require knowledge on the role of the essential gene(s) to the insect's life cycle and their expression pattern (Singh et al., 2019). Several genes are expressed at different developmental stages of an insect life cycle, some of which may be transient or stable throughout the insect life cycle. Targeting genes that are stably expressed throughout the insect lifecycle is vital to RNAi design efficiency. The availability of annotated full genome and transcriptome sequence data will be a major advantage and a step in designing RNAi constructs targeting multiple gene families for optimal efficiency. A highly complete genome assembly of *P. nigronervosa* and its symbiotic bacteria *Buchnera aphidicola* and *Wolbachia* was recently published (Mathers, Mugford, Hogenhout, & Tripathi, 2020). The availability of the genome sequence of banana aphid would help to decipher the RNAi pathway gene(s). Sequences of model aphid species such as pea aphid, *Acyrthosiphon pisum* within the same Hemiptera family, are also available in the International Aphid Genomics Consortium (Jaubert‐Possamai et al., 2007).

Silencing midgut genes like *Rack1* in green peach aphid reduced the growth of gut cells and subsequently decreased nutrient uptake (Pitino, Coleman, Maffei, Ridout, & Saskia, 2011). The work of Mulot et al. (2016) confirmed that Aly‐mRNA is one of the potential virus receptors involved in the polerovirus transmission, abundant in the whole aphids and gut cells. The gut cells were more responsive to silencing after oral feeding than other parts of the aphid body. Also, fatty acids stored as triacylglycerols serve as sources of energy and are one of the adaptive features for the survival of aphids under cold temperatures (Hubhachen, Madden, & Dillwith, 2018). Gao et al. (2019) reported that seven genes related to fatty acid synthesis pathway were upregulated in parasitized *Aphis gossypii*.

The salivary glands of aphid host a wide range of enzymes such as amylases, pectinases, cellulases, and proteases that promote its adaptive features on host plants (Cooper, Dillwith, & Puterka, 2010). The salivary secretions produced during the feeding of aphids on a plant may help in degrading the plant cell wall to facilitate penetration of the stylet or proboscis, digestion of phloem nutrients like carbohydrates and breakdown or detoxification of defensive compounds (polyphenol oxidase, peroxidase, and oxidoreductase) produced by plants (Van Bel & Will, 2016; Boulain et al., 2018; Cooper, Dillwith, & Puterka, 2011; Darvishzadeh, Bandani, & Mousavi, 2014). Mutti et al. (2008) identified a salivary effector protein, C002, in pea aphid as an essential protein for aphid–plant interaction, which can be used as a candidate gene for RNAi. C002 was found to be crucial in the feeding and survival of pea aphid on fava bean and suppresses plant defenses by detoxifying plant induced secondary metabolites like phenols (Mutti et al., 2008). Further, Mp10 and Mp42 were reported to suppress the reproductive potential of aphids and as well as trigger plant defenses (Bos et al., 2010). Two endosymbiotic microorganisms, *B. aphidicola* and *Wolbachia* sp., present in the insect's hemolymph, plays important role in the reproduction and survival of *P. nigronervosa* (De Clerck et al., 2015). GroEL, a bacterial protein found in the watery saliva of aphids, originates from *B. aphidicola* seems to induce plant defense responses (van Bel & Will, 2016). Arabidopsis plants over‐expressing *groEL* demonstrated enhanced resistance against the green peach aphid (*Myzus persicae*; Chaudhary, Atamian, Shen, Briggs, & Kaloshian, 2014). Similarly, angiotensin‐converting enzyme (ACE1 and ACE2) identified in the saliva of pea aphid is essential for its feeding and survival (Wang, Dai, et al., 2015; Wang, Luo, et al., 2015). Other enzymes such as MIF1, Armet, ACYPI39568 (a cysteine‐rich protein), and glutathione *S*‐transferase 1 are also reported to be essential for feeding and survival of plant aphids (Guo, Song, et al., 2014; Guo, Wang, et al., 2014; Kang et al., 2019; Naessens et al., 2015; Wang, Dai, et al., 2015; Wang, Luo, et al., 2015; Zhang et al., 2018).

Alkaline phosphatase is widely distributed in insect's alimentary canal, storage tissue, reproductive system, and glands (Day, 1948). It is the salivary enzyme, first identified in saliva of a Russian wheat aphid involved in aphid penetration and feeding mechanism (Cooper et al., 2011). Silencing the expression of salivary sheath protein (shp) required for the ingestion of phloem sap led to decreased growth, fecundity, and survival of grain aphid (Abdellatef et al., 2015). Further work on grain aphid revealed several categories of salivary proteins, including calcium ion binding proteins, odorant‐binding proteins, effector inducing or suppressing plant defenses to digestive, and detoxifying enzymes. Some examples of protein transcripts include beta‐mannosidase, which helps in degrading the cell wall to enhance probing and feeding by aphids, cytochrome oxidases, glutathione S‐transferases 1, esterase FE4 and esterase E4 are responsible for degrading toxic secondary metabolites expressed by the plant to modulate defense mechanisms upon insect attack. Beta‐glucosidase is an effector that initiates plant defense responses, and glucose oxidase is known to suppress plant defense, regucalcin, reticulocalbin, and calumenin are identified calcium ion binding protein that interferes with the signaling pathway of inducing plant defense response (Zhang et al., 2018). Besides salivary protein, which has been studied extensively, other enzymes in the aphid may be targeted by RNAi to inhibit aphid's viral transmission capacity. Lipids and fatty acids such as palmitic, stearic, and oleic acids are crucial to the biology of insects (Stanley‐Samuelson, Jurenka, Cripps, Blomquist, & Renobales, 1988). Alterating or shutting down of the biochemical pathway of fatty acid production may reduce aphid's fecundity or impact mortality. Gao et al. (2019) observed an increase in the fatty acid content of parasitized cotton aphid in the early stage of parasitization and a decrease after three days of parasitization. Also, disruption on the function of fatty acid desaturase 7 (FAD7), a common naturally occurring desaturase in plants, in *spr2* mutant tomato conferred resistance to potato aphid and a mutation in the FAD7 gene in *Arabidopsis thaliana* conferred resistance to green peach aphid (Li, Avila, Tieman, Klee, & Goggin, 2018). Oxidation of fatty acids, such as oxylipins, is responsible for the resistance to the Russian wheat aphid (Berner & Van Der Westhuizen, 2015). Nalam, Keeretaweep, Sarowar, and Shah (2012) identified that LOX5‐synthesised oxylipins increased the infestation of green peach aphids on *Arabidopsis* foliage.

Metabolism of lipids is associated with insect flight muscles (Haunerland, 1997). Silencing of the lipid metabolism genes (3‐ketoacyl‐CoA thiolase, phosphoenolpyruvate carboxykinase, and glycogen phosphorylase‐like isoform 2) via RNAi impacted wing development in a citrus aphid (Shang et al., 2016). The abnormal wing disk (*awd1* and *awd2*) genes encoding a nucleoside diphosphate kinase were significantly expressed in wingless than in winged morphs in *A. gossypii* and identified to play a significant role in the development and differentiation of insects (Yang et al., 2014). Loss of function of the *Drosophila awd* caused lethality in *Drosophila* (Yang et al., 2014). This explains the genetic basis of the significance of lipids and fatty acids alike in mobility and dispersal of aphids.

Acetylcholinesterase, a serine hydrolase that regulates acetylcholine, occurs in insects, mammals, and birds. It is sensitive to anticholinesterase compounds such as the organophosphate and carbamate insecticides for controlling agricultural pests (Dou et al., 2013; Fremaux et al., 2002; Soreq & Seidman, 2001). However, the impact of the insecticide residues is not environmentally friendly, potentially effecting an off‐target organism. The selective and targeted inhibition of this enzyme in aphids through the RNAi approach can selectively control insect pests without adverse environmental impact. RNAi was successfully used to silence the expression of *acetylcholinesterase* (MpAChE2) gene in the green peach aphid, and transgenic plants were resistant to aphids (Guo, Song, et al., 2014; Guo, Wang, et al., 2014).

Efficacy of dsRNA has also been linked to the mode of uptake of dsRNA into insect pest which could be through injection, ingestion, or a recently modified topical application of dsRNA using nanoparticle technology to form a stable and sustained dsRNA delivery to protect plants against insect pests and has been applied to green peach aphid (Basnet & Kamble, 2018; Knorr et al., 2018; Mitter et al., 2017; Worrall et al., 2019). This new approach of topical spray of dsRNAs targeting the coding region of the potyviral nuclear inclusion b (Nib) protein and CP enabled the inhibition of *Bean common mosaic virus* (BCMV) transmission by aphids. The study indicated that a 5‐day spray of BCMV‐CP dsRNA on the plants successfully protected *Nicotiana bentamiana* and *Vigna unguiculata* plants from infection when exposed to viruliferous aphids (Worrall et al., 2019). The use of nanoparticle spray technology could serve as an innovative paradigm for managing BBTV and eradicating BBTD. The topical application of dsRNA is cost and time effective and can be used as a non‐GM‐based approach for plant protection against insect pests.

## 8 CONCLUSION

**FIGURE 3:**
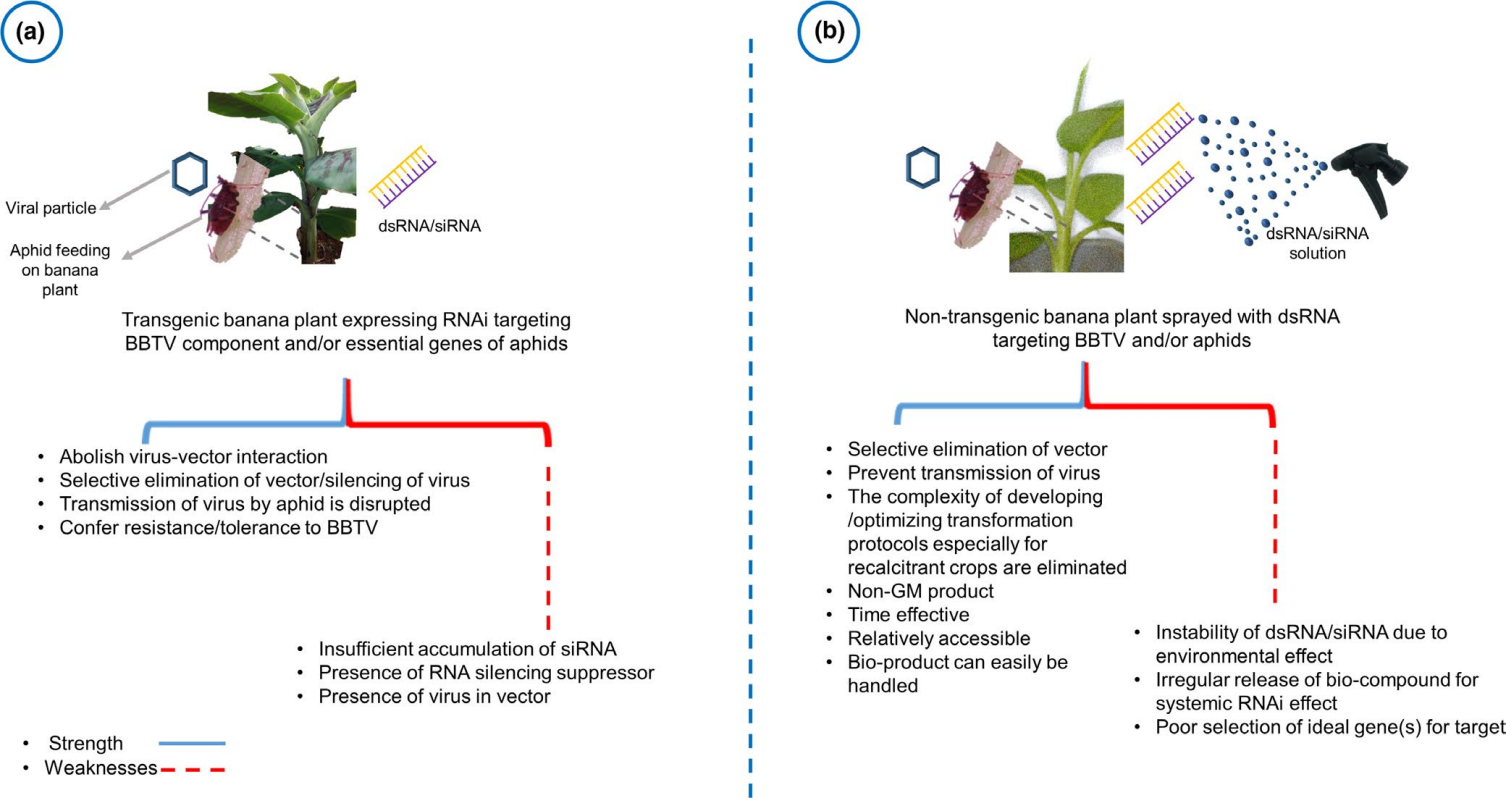
A representation of different approaches of RNAi delivery for control of the banana bunchy top disease. (a) Transgenic banana expressing RNAi targeting BBTV and/or aphids transmitting the virus, (b) nontransgenic approach of dsRNA topical spray for control of *Banana bunchy top virus* and/or aphids

The advances in biotechnological approaches to inhibit the destructive potentials of pests and diseases open up avenues for crop improvement. BBTD is becoming a global pandemic in banana and plantain producing regions, if not controlled. The attributed huge production losses intimate a significant threat to banana and plantain production, which is a major crop that sustains smallholder farmers and contributes to the annual income of several countries in the world. Managing the spread of vector‐borne plant diseases is a sustainable way to increase crop production. The control approach will depend on the intended outcome, which could either suppress banana aphid's population or inhibit its ability to transmit BBTV, causing BBTD. RNAi is a promising strategy, which can control both the virus and insect vector transmitting the virus. The delivery of RNAi into the host plants can be transgenic or nontransformative alternatives that are topically applied dsRNA (Figure 3). The effectiveness of RNAi technology depends on the specificity of the homologous mRNA sequence to the target genes, the stoichiometry of siRNA accumulation while restricting the influence of RNA‐silencing suppressors. Simultaneous expression of ideal genes within the same family or multiple families through gene pyramiding could be a promising approach to induce systemic RNAi effect on *P. nigronervosa* and BBTV. Therefore, it is imminent that a holistic approach required for control of the spread of BBTD through the use of RNAi‐based technology is feasible.

## CONFLICTS OF INTEREST

The authors declare no conflict of interest.
